# Critical policies disparity of the first and second waves of COVID-19 in the United Kingdom

**DOI:** 10.1186/s12939-022-01723-3

**Published:** 2022-08-22

**Authors:** Manfei Yang, Leiyu Shi, Haiqian Chen, Xiaohan Wang, Jun Jiao, Meiheng Liu, Junyan Yang, Gang Sun

**Affiliations:** 1 Department of Health Management, School of Health Management, Southern Medical University, Guangdong 510515 Guangzhou, China; 2 Department of Health Policy and Management, Bloomberg School of Public Health, Johns Hopkins University, Baltimore, MD 21205 USA

**Keywords:** COVID-19, Critical policies disparity, The first and second waves, Prevention and control policies, Mitigation strategy

## Abstract

**Objective:**

This study aims to compare the differences in COVID-19 prevention and control policies adopted by the United Kingdom (UK) during the first wave (31 January 2020 to 6 September 2020) and the second wave (7 September 2020 to 12 April 2021), and analyze the effectiveness of the policies, so as to provide empirical experience for the prevention and control of COVID-19.

**Methods** We systematically summarized the pandemic prevention and control policies of the UK from official websites and government documents, collated the epidemiological data from 31 January 2020 to 12 April 2021, and analyzed the effectiveness of the two waves of pandemic prevention and control policies.

**Results:**

The main pandemic prevention and control policies adopted by the UK include surveillance and testing measures, border control measures, community and social measures, blockade measures, health care measures, COVID-19 vaccination measure, and relaxed pandemic prevention measures. The new cases per day curve showed only one peak in the first wave and two peaks in the second wave. The number of new cases per million in the second wave was much higher than that in the first wave, and the curve fluctuated less. The difference between mortality per million was small, and the curve fluctuated widely.

**Conclusion:**

During the first and second waves of COVID-19, the UK implemented three lockdowns and managed to slow the spread of the pandemic. The UK’s experience in mitigating the second wave proves that advancing COVID-19 vaccination needs to be accompanied by ongoing implementation of non-pharmacological interventions to reduce the transmission rate of infection. And a stricter lockdown ensures that the containment effect is maximized during the lockdown period. In addition, these three lockdowns featured distinct mitigation strategies and the UK’s response to COVID-19 is mitigation strategy that reduce new cases in the short term, but with the risk of the pandemic rebound.

## Introduction

In December 2019, the first confirmed case of the Coronavirus disease 2019 (COVID-19) caused by Severe Acute Respiratory Syndrome Coronavirus 2 (SARS-CoV-2) appeared in Wuhan, Hubei Province, China. Since then, the COVID-19 pandemic has spread rapidly around the world. On 31 January 2020, World Health Organization (WHO) declared COVID-19 a global “pandemic” and a public health emergency of international concern [1]. The COVID-19 pandemic has exposed all of humanity to an unprecedented public health crisis, which has had a huge impact on global economic development, social stability, and public health governance. As of August 2022, the cumulative confirmed COVID-19 cases exceeded 500 million and the cumulative deaths exceeded 6 million worldwide [2], and although SARS-CoV-2 is significantly less lethal than SARS, the spread is much greater.

Since the global pandemic of COVID-19, governments have formulated policies based on national conditions to mitigate or contain the spread of the pandemic. Non-pharmacological interventions (NPIs) were the most important means of addressing COVID-19 during the period when the COVID-19 vaccine was not in use and vaccine coverage was low, such as lockdowns, quarantine of patients, travel restrictions, and cancellation of gatherings, with the aim of reducing the spread of the virus as well as the size of the epidemic peak to gain a buffer for the development and use of vaccines and specific drugs [3]. With the successful development, evaluation, and production of multiple COVID-19 vaccines, vaccination was gradually added to prevention and control policies aimed at reducing the rate of COVID-19 infection by protecting susceptible populations.

The United Kingdom (UK) is currently one of the countries most affected by the COVID-19 pandemic. By December 2021, the cumulative confirmed cases in the UK have exceeded 10 million, ranking first in Europe, and the cumulative deaths have exceeded 1.4 million [4]. England, Scotland, Wales, and Northern Ireland have all responded to the pandemic. Since England accounts for 80% of the total UK population and most confirmed cases in the UK were found in England, England’s policy has the greatest impact on the overall pandemic prevention and control policy of the UK [5]. In response to COVID-19, the UK government has adopted a mitigation strategy, or “flu pandemic-like strategy”. They concluded that the spread of COVID-19 cannot be completely stopped, but only by taking more moderate measures to slow down its spread and flatten the pandemic curve until the population forms an adequate immune barrier, COVID-19 becomes a seasonal infectious disease such as influenza [6]. Therefore, the pandemic prevention and control policies adopted by the UK, such as lockdown, are characterized by a clear mitigation strategy.

In December 2020, the UK approved the Pfizer-BioNTech vaccine and initiated vaccination, and by December 2021, 89% of the UK population had received the first dose and 81% had received the second dose [4].

This study systematically summarized and analyzed the prevention and control policies adopted by the UK during the first and second waves of COVID-19, in order to provide empirical experience for other countries and regions in the world that are combating COVID-19.

## Methods

### Data collection

Epidemiological data of COVID-19 were extracted from Johns Hopkins University & Medicine Coronavirus Resource Center [7], the Office for National Statistics (ONS) [8], and Worldometers Website [2]. Indexes including daily new cases, daily deaths, cumulative confirmed cases, cumulative deaths, mortality per million, new cases per million. Data were collected from 31 January 2020 to 12 April 2021, in which the first wave was from 31 January to 6 September 2020, and the second wave was from 7 September 2020 to 12 April 2021.

The first confirmed case of COVID-19 occurred in the UK on January 31, 2020, which is the start date of the first wave of COVID-19. On September 7, 2020, the number of new cases per day in the UK exceeded 3000, the highest number in nearly 4 months. Therefore, we set September 7, 2020 as the start date of the second wave of the pandemic, and the day before it was the end date of the first wave. After implementing a series of policies, the UK officially entered the second phase of lockdown on April 12, 2021, which is defined as the end date of the second wave. Since then, the UK has ushered in a new peak of the pandemic.

### Policies information

The prevention and control policies were selected from official government websites, prime minister’s TV speeches and official government documents, such as National Health Services (NHS) [9], Department of Health and Social Care Website [10]_._ The policies were mainly summarized as surveillance and testing measures, border control measures, community and social measures, blockade measures, health care measures, COVID-19 vaccination measure, and relaxed pandemic prevention measures.

Finally, we collated and compared the first and second waves of pandemic prevention and control policies. At the same time, we drew the epidemiological timeline of two waves, and combined the key policies of each wave with new cases per million to evaluate the effectiveness of the COVID-19 strategy adopted by the UK.

## Results

### Major prevention and control policies during two waves

#### The first wave

Compared to Asian countries such as China and South Korea, the peak of COVID-19 infections in the UK emerged later, so the UK did not adopt strict prevention and control measures in the early stage, but funded research and development of COVID-19 vaccine and established COVID-19 screening centers. In March 2020, the UK published its anti-pandemic plan, including four stages of Contain, Delay, Research, and Mitigate. And announced on 12 Mar 2020, the UK moved from the “contain” stage to the “delay” stage of tackling the spread of coronavirus. The day before the announcement of the “delay stage”, the Bank of England (BOE) announced its stimulus package, lowering the benchmark interest rate in response to the impact of COVID-19 on the economy and to prevent the short-term difficulties caused by the pandemic from having a lasting negative impact. In response to the continuing high level of COVID-19, on March 16, 2020, the government issued a “risk aversion” policy, which focused on advising and urging people to go out less, avoid gathering, and expand their social distance, but did not mandate its implementation. In the period leading up to the March 20, 2020, closure of schools and cancellation of exams in the UK, the focus of the UK’s prevention and control was on maintaining socioeconomic stability and relieving pressure on the healthcare system during the surge in COVID-19 cases. From 23 March to 7 May 2020, the UK imposed its first lockdown, during which measures were stricter and more extensive than in the early stage of the pandemic, seeking to ease the pressure of the peak and flatten the epidemic curve through extended social distances, care of critically ill patients, and conscious isolation of the population. When the pandemic began to decline in mid-May 2020, the UK government announced the lifting of the lockdown. Table 1 shows the major prevention and control policies adopted by the UK during the first wave of the pandemic.

**Table 1:** Major pandemic prevention and control policies in the UK during the first wave^a^

Policy	The key elements
Surveillance and testing measures	(1) The COVID-19 Drive-Through Screening Centre was set up at Parsons Green Health Centre and Edinburgh Western General Hospital on 24 and 28 Feb 2020 respectively. (2) The UK incorporated COVID-19 testing for severe acute respiratory illness (SARI) and ILI surveillance. (3) Since mid-April 2020, the UK has expanded the range and capacity of Coronavirus testing. (4) In May 2020, the NHS released a test version of Coronavirus contact tracking app. (5) At the end of May 2020, the Coronavirus detection and tracking system was activated in England and Scotland.
Border control measures	(1) In March 2020, the UK went into lockdown. The government banned all non-essential travel. (2) On 8 June 2020, the UK introduced an entry quarantine policy, requiring passengers entering the UK to be quarantined for 14 days. (3) On 28 July 2020, the UK imposed a 14-day quarantine on people arriving from Spain.
Community and social measures	(1) In March 2020, the government introduced a “risk aversion” policy, avoiding non-essential outdoor and personal contact, quarantining those with symptoms at home for 14 days and working from home, a social distancing measure of 2 m. (2) On 20 March 2020, all schools in the UK were closed and all tests cancelled. And indefinitely close bars, restaurants, cafes, gyms, cinemas and other public places across the country. (3) People have been asked to wear masks on public transport.
Blockade measures	(1) On 11 May 2020, the Prime Minister proposed a level 5 alert system as the basis for judging the UK’s lockdown phase. (2) On 23 March 2020, the UK entered into a three-week national lockdown. All people were asked to stay at home, do not go out as far as possible, prohibited public gatherings of more than two people. (3) On 17 April 2020, the lockdown was extended by 3 weeks until 7 May. (4) On 6 August, the UK imposed a second lockdown on sites in Scotland where there have been clusters of coronavirus infections.
Health care measures	(1) The NHS cancelled all non-emergency operations and sent as many patients home as possible to free up 30,000 beds in England. (2) Temporary intensive care hospitals were established in various regions, the NHS Louisa Jordan was established in Scotland, temporary critical care NHS Nightingale hospitals were built across England, and the Dragon’s Heart Hospital was set up in Wales. (3) Primary care practitioners were advised to avoid face-to-face assessment of suspected cases.
COVID-19 vaccination measure	On 3 February 2020, the UK announced a £20 million investment to accelerate the development and production of a COVID-19 vaccine.
Relaxed pandemic prevention measures	(1) On May 11th the prime minister announced a gradual easing of the blockade in three stages, with “conditional” lifting. (2) On 21 May 2020, Scotland announced a four-stage initial unblock plan. (3) On 26 May 2020, new retail unblock guidelines were published and all non-essential retail outlets are expected to reopen in the UK from 15 June. (4) The COVID-19 alert level was lowered from phase 4 to phase 3 on 19 June 2020. (5) On 4 July 2020, cinemas, libraries, restaurants and bars across England were opened to the public. Social distancing can be reduced to 1 m if people wear masks and other protective measures.

^a^The first wave was from 31 January to 6 September 2020

#### The second wave

In the second wave, the UK government adopted stricter prevention and control policies. On October 12, 2020, England had different requirements for “medium”, “high” and “very high” risk areas. Indoor and outdoor gatherings of more than six people were banned in medium-risk areas, bars and restaurants were limited to open for a limited time. Banned parties in high-risk areas and reduce travel; Very high risk areas, banned parties and banquets, closed bars. Shops, schools and colleges remained open across England. It is worth mentioning that Scotland, Wales and Northern Ireland all started to implement their own pandemic prevention measures from October 2020 when England was in full lockdown in early November 2020. In addition, Premier League was held in the UK during the period of total lockdown. In December 2020, the UK approved the Pfizer-BioNTech vaccine, becoming the first country in the world to do so. Since then, England raised the level of quarantine and imposed the third lockdown. Thanks to the strict third lockdown, the pandemic improved and on March 29, 2021, the UK entered the first phase of lifting the lockdown; on April 12, it entered the second phase of lifting the lockdown as planned. However, the UK did not proceed to the third and fourth stages of lifting due to a new peak of the pandemic. Table 2 shows the major prevention and control policies adopted by the UK during the second wave of the pandemic.

**Table 2:** Major pandemic prevention and control policies in the UK during the second wave^a^

Strategy	The key elements
Testing measures	In April 2021, the UK government launched a universal free COVID-19 testing program, offering free rapid COVID-19 testing to all citizens twice a week.
Border control measures	(1) From 11 January 2021, all passengers arriving in the UK on international flights, ships and trains were required to show proof of a negative nucleic acid test within 72 hours of departure when entering the UK. (2) All travelers from countries outside the Government’s Travel Corridor List were required to self-quarantine for 10 days upon arrival in the UK. (3) All travelers were required to complete a passenger location form and be subject to lockdown within 48 hours of arrival in the UK.
Community and social measures	(1) Gatherings of more than six people were banned in England in mid-September 2020. (2) By the end of September 2020, bars and restaurants were operated on limited hours, and the wearing of face masks was extended to retail, cab and hotel staff, with fines for violators. (3)The three-tier alert system for England was announced, with “medium”, “high” and “very high” levels, each corresponding to different levels of severity and response. (4)In December 2020, the self-quarantine period for close contacts was reduced from 14 days to 10 days.
Blockade measures	(1) On 14 October 2020, the UK implemented tier-three lockdown measures, with different levels of restrictions in different areas depending on the severity of the coronavirus outbreak. (2) On 5 November 2020, the second lockdown in 4 weeks was imposed in England, with schools remaining open, non-essential shops and gyms closed and people staying at home without special needs. An “enhanced” three-tier alert system was implemented since the lockdown ended. (3) On 19 December 2020, the Christmas reunion programme was cancelled, and all areas of the south East of England in Tier 3 were upgraded to Tier 4, the strictest level. (4) On 5 January 2021, the third round of COVID-19 lockdown measures was launched, requiring people to stay at home until mid-February, not to go out unless necessary, and to close all schools and most shops.
COVID-19 vaccination measures	(1) The MHRA gave regulatory approval to the Pfizer–BioNTech vaccine on 2 December 2020. (2) Vaccination began on 8 December 2020 in 70 hospitals within the country with storage conditions. (3) On 30 December 2020, the MHRA approved the Oxford-AstraZeneca vaccine for use in a two-dose schedule. (4) Delayed the second dose of Pfizer–BioNTech vaccine and extended the interval between the two doses to 12 weeks, allowing for more people to be immunised with a first dose. (5) The government developed mass vaccination centres, staffed by trained health workers. (6) In order to improve access in disadvantaged communities, there have been vaccination sites in religious buildings such as churches and mosques as well as “pop-up” and mobile sites.
Relaxed pandemic prevention measures	(1) In early December 2020, the lockdown was fully lifted and most of England was classified into phase 2 and 3. (2) On 22 February 2021, four steps to relax pandemic containment measures in England. (3) On 29 March 2021, the UK entered the first phase of lifting the lockdown, allowing outdoor gatherings of fewer than six people, opening up outdoor sports fields and restoring outdoor team sports. s(4) On 12 April 2021, the UK moved into phase two to lift lockdown, allowing outdoor meals for more than six people, opening entertainment venues, allowing outdoor dining, allowing travel between England and Wales, opening driving schools and relaunching driving tests.

^a^The second wave was from 7 September 2020 to 12 April 2021

### Epidemiological trends of two waves

#### The first wave

**Fig. 1:**
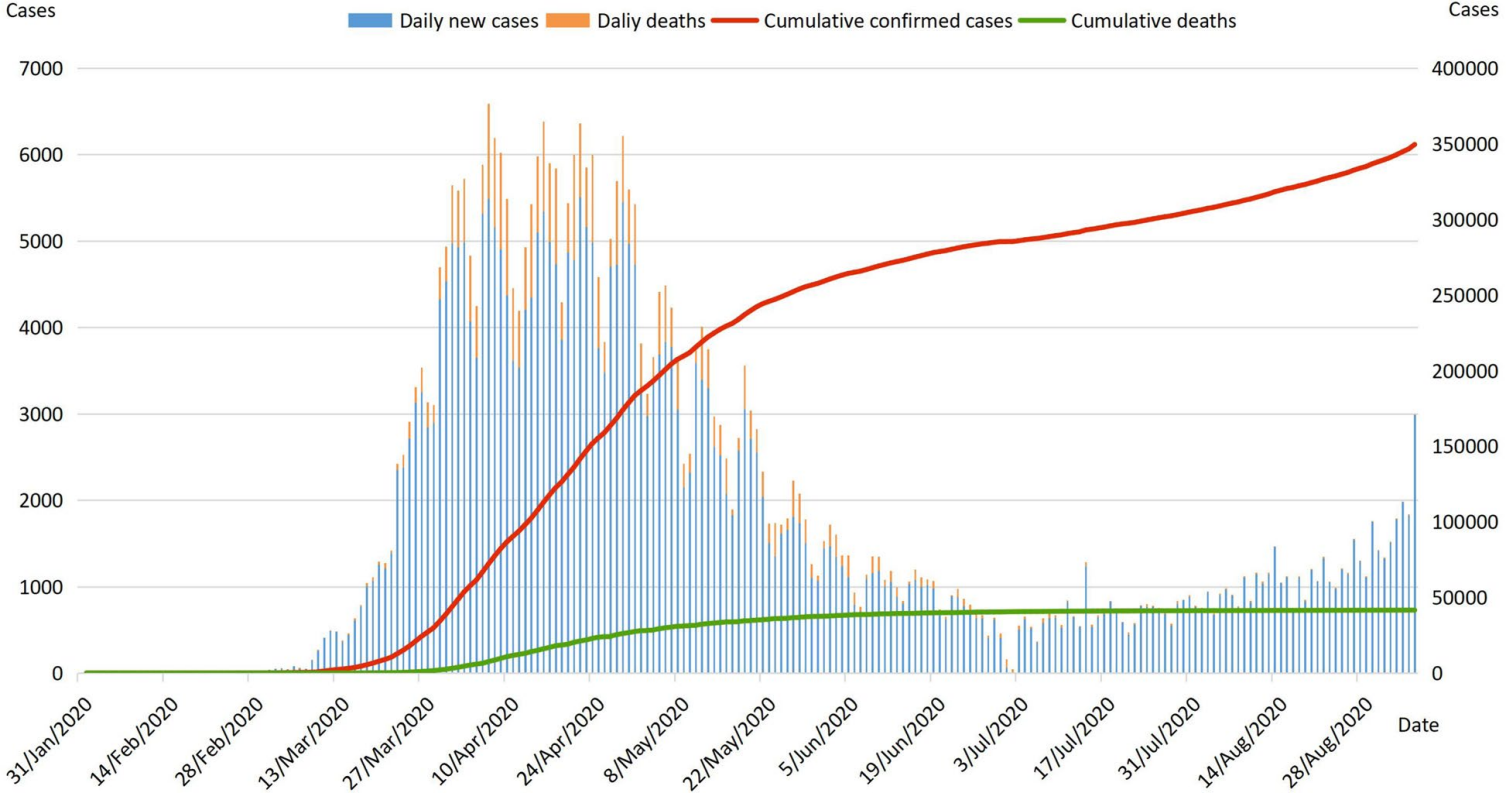
Epidemiological timeline of the first COVID-19 wave in the United Kingdom. Note: Daily new cases and daily deaths refer to main axis (left). Cumulative confirmed cases and cumulative deaths refer to secondary axis (right)

From the first case of COVID-19 on January 31, 2020 until the end of February 2020, the pandemic in the UK was in a slowly growing and relatively stable state. The day after WHO declared COVID-19 a pandemic, Thursday was also the first time the day-on-day increase reached three figures in the UK [11]_._ Since mid-March 2020, the pandemic gradually got out of control in European countries such as Italy and Spain, and the UK was affected, with a significant increase in the number of daily new cases and a marked rise in COVID-19 transmission. From the end of March to the end of April, the number of daily new cases exceeded 3000 almost every day, and the cumulative confirmed cases curve increased steeply. The number of daily new cases on April 22 was the highest in the first wave, at more than 5500. As a result, the UK imposed its first nationwide lockdown on March 23. Subsequently, the number of daily new cases and daily deaths gradually decreased, the cumulative deaths curve flattened, the transmission and hospitalization rate also decreased [12]. The percentage of people testing positive for COVID-19 rose slightly in July after a low in June, and then held steady for some time. Figure 1 shows the epidemiological timeline of the first COVID-19 wave in the United Kingdom.

#### The second wave

**Fig. 2:**
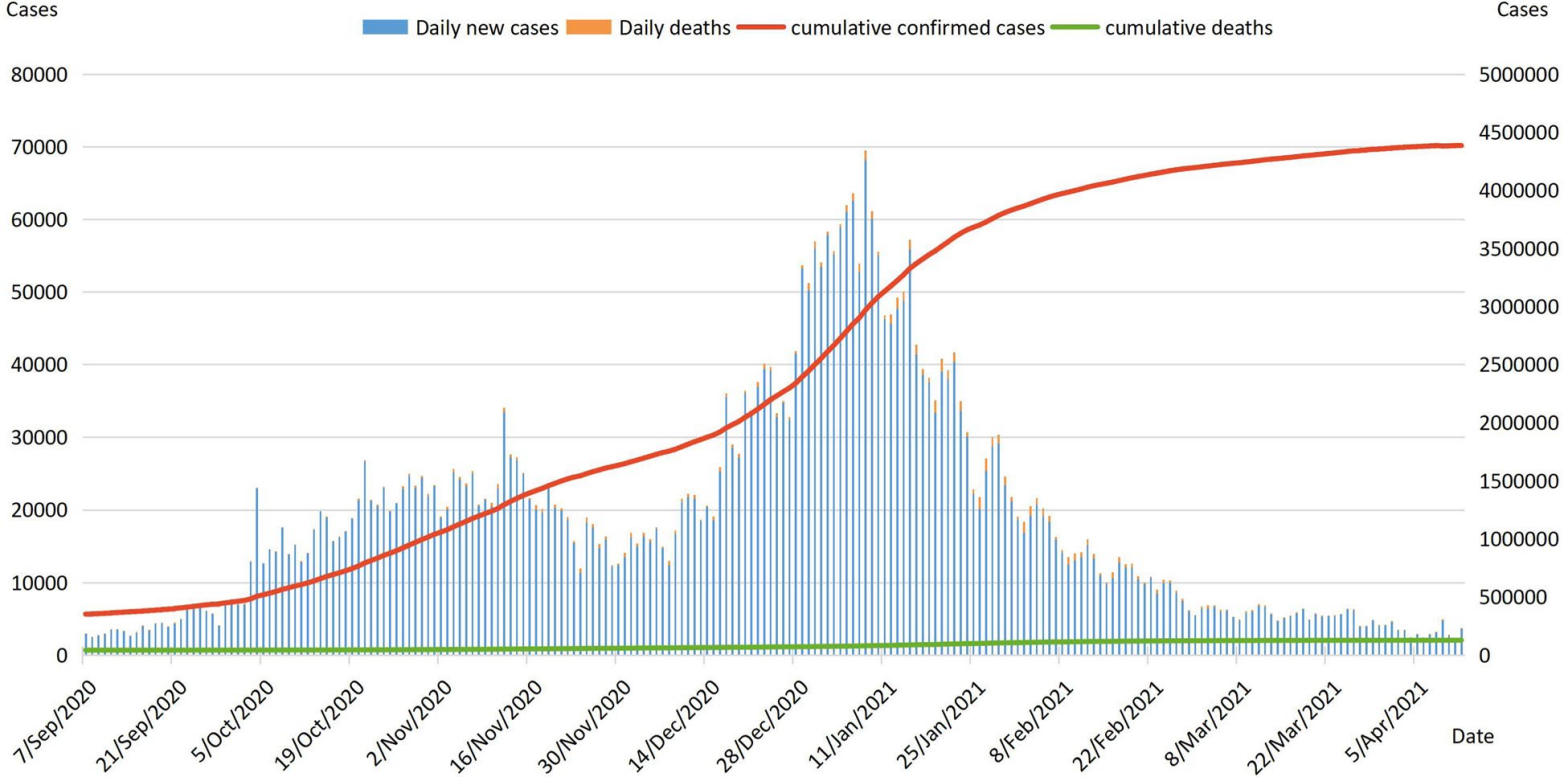
Epidemiological timeline of the second COVID-19 wave in the United Kingdom. Note: Daily new cases and daily deaths refer to main axis (left). Cumulative confirmed cases and cumulative deaths refer to secondary axis (right)

The second COVID-19 wave in the UK began on September 7, 2020, a day when the number of daily new cases exceeded 3000, the highest since the lockdown was lifted in May 2020. The number and incidence of COVID-19 infections both increased in the first week of September 2020, with higher rates in northwest England and London [13]. In October 2020, confirmed cases increased rapidly and cumulative confirmed cases curve showed a significant upward trend. As a result, England started the second national lockdown in early November. After a four-week lockdown, the number of confirmed cases began to decline in late November 2020. But schools in the UK remained open during the second lockdown, so the infection rate of primary school-aged children, secondary school-aged children, older teenagers and young adults remains high [14]. The number of daily new cases increased sharply since mid-December 2020. Between 29 December 2020 and 10 January 2021, the number of daily new cases exceeded 50,000, with 4 days of daily new cases exceeding 60,000. As a result, the UK government cancelled the Christmas reunion program, raised the level of quarantine and started the third national lockdown. The number of infections continued to rise until the end of January 2021, after which the pandemic curve flattened out and the UK gradually entered the phase of lifting the lockdown. In the second COVID-19 wave, most of the confirmed cases were concentrated in London, so the UK government implemented a series of measures for England. Figure 2 shows the epidemiological timeline of the second COVID-19 wave in the United Kingdom.

#### Comparison of epidemiological curves between the first and second waves

**Fig. 3:**
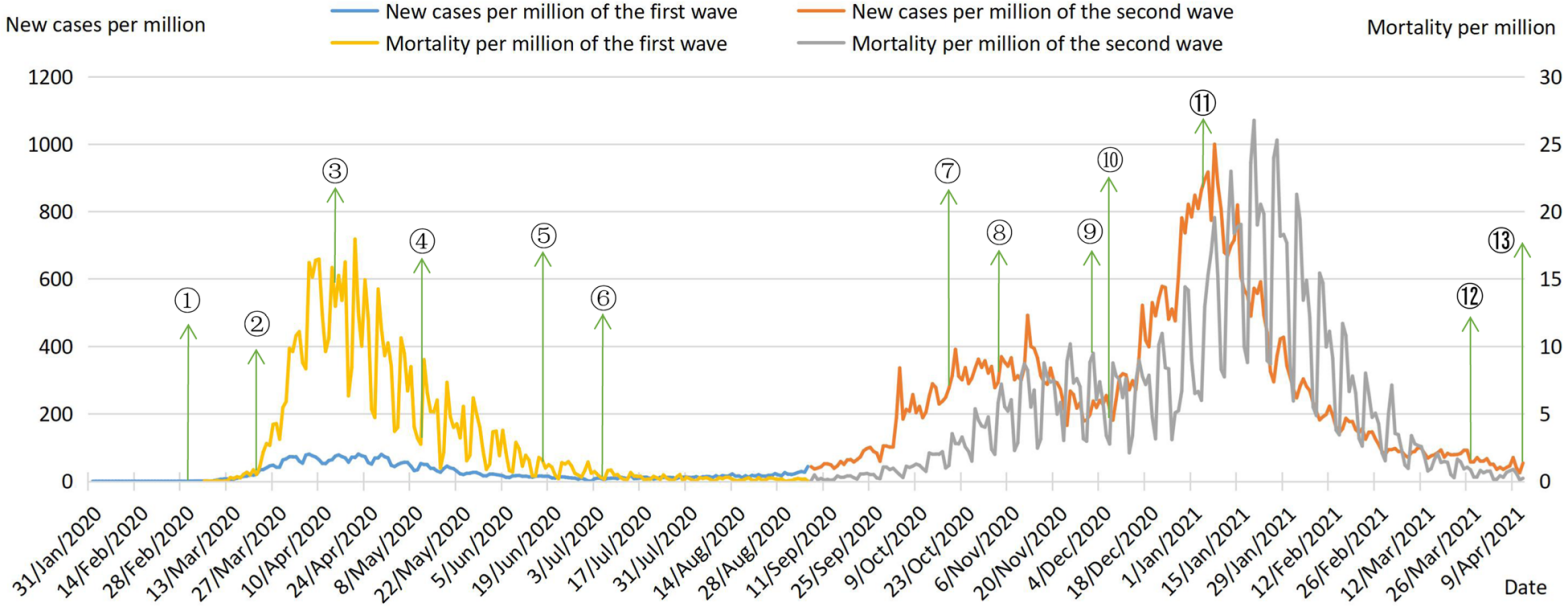
Curves of new cases per million and mortality per million of two COVID-19 waves. Note: New cases per million of the first wave and New cases per million of the second wave refer to main axis (left). Mortality per million of the first wave and Mortality per million of the second wave refer to secondary axis (right). ①. On 3 Mar 2020, the UK launched a four-stage plan to combat the COVID-19;②. On 23 Mar 2020, the UK entered into a three-week national lockdown;③. On 17Apr 2020, the lockdown was extended;④. On 11 May 2020, a gradual easing of the blockade in three stages was announced;⑤. On 19 June 2020, the COVID-19 alert level was lowered;⑥. On 7 Jul 2020, massive lockdown lifting across England;⑦. On 14 Oct 2020, a tier-three lockdown was implemented;⑧. On 5 Nov 2020, the second lockdown in four weeks was imposed in England;⑨. On 2 Dec 2020, the lockdown was fully lifted; Pfizer-BioNTech vaccine was approved;⑩. On 8 Dec 2020, vaccination started;⑪. On 5 Jan 2021, the third round of COVID-19 lockdown measure was launched;⑫. On 29 Mar 2021, the UK entered the first phase of lifting the lockdown;⑬. On 12 Apr 2021, the UK moved into phase two to lift lockdown

The criteria for COVID-19 infection in the UK is that swabs taken from the nose and throat test positive for SARS-CoV-2 regardless of the presence of symptoms. Based on official data, we calculated new cases per million and mortality per million of the first and second waves. Figure 3 shows curves of new cases per million and mortality per million of the first and second COVID-19 waves and key policies. It is obvious that the number of new cases per million in the second wave is much higher than that in the first wave, and the fluctuation range of the curve in the second wave is also larger than that in the first wave. In the first wave，the highest value of new cases per million was 80.71 on April 22, 2020. New cases per million were greater than 50 from 30 March to 1 May 2020, with the largest increase on April 6, rising to 77.939 from 53.513 the day before. In the second wave, the highest value of new cases per million was 999.778 on January 8, 2021, approximately 12 times the highest value in the first wave. New cases per million from 28 December 2020 to 16 January 2021 were above 600, with the largest increase on January 8, 2021, to 999.778 from 773.922 the previous day.

The main reason for more new cases per million in the second wave was thatin late December 2020, Alpha variant began to spread massively in the UK, followed by the Beta, Gamma and Delta variants. These variants enhanced transmissibility and virulence, led to a new peak of the pandemic in the UK. In addition, during the second wave the UK improved COVID-19 testing capacity, and the government offered free COVID-19 testing to citizens, so the number of confirmed cases increased.

It is not difficult to see that the difference of mortality per million between the first and second waves is not as huge as the new cases per million, and the fluctuation range of the curve in two waves are relatively large. In the first wave, the highest value of mortality per million was 17.945 on April 21,2020. In the second wave, the highest value of mortality per million was 26.771 on January 20,2021. There was an increase in hospital and care homes deaths and a continuing rise in home deaths in the two waves of COVID-19, and there were differences in patterns of mortality among England、Scotland、Wales and Northern Ireland [15].

## Discussion

### The core policy during the first wave

In response to the first COVID-19 wave, the UK implemented a series of measures centered on the national lockdown policy to flatten the pandemic curve. On March 23, 2020, when the lockdown first implemented in the United Kingdom, new cases per million and mortality per million were 34.439 and 1.114, respectively. In April 2020, there was a significant increase in both new cases per million and mortality per million, so the UK government extended the original 3-week lockdown to May 7, 2020. After a 6-week lockdown, the UK passed the peak of the first COVID-19 wave and was back to a manageable state when the Prime Minister announced the gradual lifting of the lockdown on May 11, 2020. Although new cases per million and mortality per million had not yet been reduced to the level before the lockdown, there had been a significant decrease and a gradual downward trend had been maintained. On July 7, 2020, when the massive lockdown was lifted, new cases per million was 8.562 and mortality per million was 0.792, both of which had been reduced to the level before the lockdown. The apparent changes in new cases per million and mortality per million indicated that the national lockdown policy implemented in the first wave was extremely effective in slowing the spread of the pandemic. During this lockdown, the UK did not test, isolate and treat all COVID-19 infected people and close contacts. However the government banned all kinds of public gatherings and social activities, closed schools, cinemas, shopping malls and other public places, and people were asked to stop all unnecessary going out. Through lockdown, the frequency of people going out is reduced and the transmission chain of the virus is effectively cut off [16]. These measures widened the distance between people and prevented the population from gathering, effectively mitigating the surge in infections and flattening the pandemic curve in the short term.

### Core policies during the second wave

During the second wave, national lockdown was the core of the pandemic prevention and control policies, and the effect of slowing down the spread of the pandemic was very obvious. In response to the rebound of the pandemic, two lockdowns were imposed in the UK, but they differed in duration, specific measures and effectiveness.

On November 5, 2020, new cases per million and mortality per million increased to 354.274 and 5.542 respectively. England started a four-week lockdown. On December 2, 2020, new cases per million were below the level at the time the lockdown was imposed, and although mortality per million was 9.5, still higher than the value before lockdown, they both trended downward. And the infection rate was reduced to a manageable level, so the UK fully lifted the lockdown. Unlike the other two lockdowns, this one was less intense and shorter in duration. Government public services and supermarkets were not closed during the lockdown, the catering industry could continue to provide delivery and takeaway services, schools remained open, and Premier League continued to be held. In addition, the UK government was aware that a prolonged lockdown will lead to an increased willingness to gather, and the increase in the number of gatherings after the lifting of the lockdown will lead to the rebound of the pandemic, while the short-term lockdown will alleviate some of this pressure. Therefore, this lockdown is mainly to implement a series of measures to alleviate the severe pandemic situation against the backdrop of the intensified global rebound of COVID-19, thus preparing for the Christmas holidays.

On January 5, 2021, the British Prime Minister announced that England was entering the third lockdown. It is worth noting that in late December 2020, the more infectious Alpha Coronavirus variant began to spread massively in the UK. As a result, new cases per million had increased to 895.61 and mortality per million had increased to 12.917, by the time the third lockdown was imposed. This lockdown was stricter than the previous two lockdowns, with England’s pandemic prevention level adjusted to the highest level of 5. All normal school instruction was shut down, only distance learning was allowed, and all exams were suspended. A strict quarantine program for high-risk susceptible populations was also reinstated, prohibiting non-essential outings for all populations. In mid-January 2021, the pandemic in the UK reached a turning point. Although new cases per million remained at a high level, it showed a downward trend. Thanks to the strict lockdown, the UK entered the first phase of lifting the lockdown on March 29, 2021. By this time new cases per million were well below the level at the beginning of the lockdown, mortality per million was lower than 0.5, and the pandemic in the UK had improved significantly.

Prior to the third lockdown, the UK had approved the COVID-19 vaccine and initiated vaccination in December 2020. Despite the rapid response to vaccination in the UK, COVID-19 in the UK has reached a new peak, with new cases per million and mortality per million both growing to new peaks. At the inflection point of the pandemic in mid-January 2021, the vaccination coverage rate of COVID-19 vaccine in the UK was less than 5%. As of April 12, 2021, when the UK entered the second phase of lifting the lockdown, the coverage rate of vaccine first dose was less than 50%, and the percentage of completed two doses was approximately 10% [17]. In addition, Alpha, Beta, Gamma, and Delta Coronavirus variants were all transmitted in the UK during this period. Although the COVID-19 vaccine provided better prophylaxis against Alpha, Beta, and Gamma variants, its effectiveness against Delta was reduced [18, 19]. In this case, it was thanks to the timely blockade and strict non-pharmaceutical interventions that the UK managed to cope with a strong rebound of COVID-19. Ideally, uptake by a large enough proportion of the population will offer protection to people who remain unimmunised, referred to as achieving “herd immunity” [20]. For COVID-19, vaccine uptake would need to be between approximately 67 and 80% to reduce spread of the disease [21]. It is crucial to delay the peak of COVID-19 while waiting for vaccine coverage to meet the “herd immunity” criteria. The implementation of non-pharmaceutical interventions has kept the pandemic in a flat state, thus delaying the peak and reducing its height. On the one hand greatly reducing the burden on the health system, on the other hand gaining time for the COVID-19 vaccine to reach high coverage [22]. Therefore, active promotion of vaccination needs to be accompanied by continued implementation of non-pharmacological interventions to reduce the transmission rate of infection until COVID-19 is eliminated [23].

### Comparison of policies between two waves

National lockdown is one of the most effective non-pharmacological interventions to delay the spread of COVID-19 [24], and in the first and second COVID-19 waves, the UK implemented three lockdown policies to survive the peak of COVID-19 in March 2020, November 2020, and January 2021, respectively. The duration and specific measures of the three lockdowns were different. Table 3 shows the major difference between the three lockdowns during the first and second waves of COVID-19 in the UK.

**Table 3:** Major difference between the three lockdowns during the first and second waves of COVID-19 in the UK

	The first lockdown	The second lockdown	The third lockdown
**Start and end dates**	March 23, 2020 to July 7, 2020.	November 5, 2020 to December 2, 2020.	January 5, 2021 to April 12, 2021.
**Waves**	The first wave.	The second wave.	The second wave.
**Whether to spread variants**	No variants.	Alpha variant.	Alpha, Beta, Gamma, Delta variants.
**Whether the lockdown was extended**	3 weeks extension from April 14, 2020.	No extension.	No extension.
**Whether schools were closed**	Closed schools, cancelled exams.	No schools were closed.	All schools instruction were closed, only allowed distance learning.
**Whether public places were closed**	Closed public places.	Government public services and supermarkets were not closed.	Closed public places.
**Whether the lockdown was lifted in stages**	Gradually lifted the lockdown from May 11, 2020.	Completely lifted the lockdown on December 2, 2020.	Lifted the lockdown in stages from March 29, 2021.

The first lockdown lasted the longest and the second the shortest. No variant strains spread during the first lockdown, variants were present and spreading during the second and third lockdowns. As of April 12, 2021, 97,45% of the variants transmitted within the UK were Alpha, 0.6% were Beta, 0.48% were Delta, and 0.11% were Gamma [25]. The second lockdown was less stringent than the other two. During the second lockdown, the UK did not close its borders and the continuation of the Premier League brought fans from all over Europe together in the UK. Meanwhile, schools, colleges and nurseries in England were not closed, so there was no significant difference in the proportion of positive tests among different age groups in the first wave, but in the second wave, the infection rate of school-age children and young people was significantly higher than that of other age groups. Moreover, mortality per million remained at a high level during the second lockdown. Studies have shown that school closures can at best reduce morbidity and mortality from COVID-19 by about 60% [26]. Although the implementation of a stricter lockdown may affect individual behavior and mental health to a certain extent, it can ensure that the containment effect is optimized during the lockdown period and the transition to a new quarantine status is smooth after the lockdown is lifted.

In addition, all three lockdowns are characterized by a clear mitigation strategy. On the one hand, the lockdown was imposed to mitigate the surge in confirmed cases, rather than completely block the spread of the virus. On the other hand, during the lockdown, the government did not isolate and treat all confirmed COVID-19 patients, but advocated for self-monitoring and isolation at home for mildly symptomatic patients and people with fever, persistent cough and other discomfort, not to go to hospitals for virus testing and treatment, and asked suspected cases and close contacts to self-isolate and not to centrally isolate and manage them. The UK is typical of countries that have adopted a mitigation strategy in response to COVID-19, focusing on treatment, isolation and detection of critical cases, with a focus on reducing transmission rates and delaying the peak of the pandemic [27]. At the same time, in order to prevent excessive pressure on the health care system, the government has taken measures to expand social distance and limit the movement of people, but has not emphasized the screening and management of patients with mild symptoms and close contacts. In March 2020, when there was a significant increase in COVID-19 infections in several European countries, the UK expected the population to form an immune barrier to COVID-19, enabling “staggered infections” and delaying the peak of COVID-19 infections to relieve pressure on healthcare facilities. The effectiveness of the pandemic prevention control in the UK shows that mitigation strategy can slow the spread of COVID-19 in the short term and successfully reduce the number of new infections. However, without isolating the source of infection and completely blocking the spread of the virus, there is a risk that COVID-19 will rebound several times when herd immunity is insufficient [16].

## Conclusion

This study systematically collated and analyzed the prevention and control policies adopted by the UK during the first and second COVID-19 waves. Based on the epidemiological data, it was found that there were significant differences in new cases per million and smaller differences in mortality per million between the two waves. The UK’s experience in mitigating the second wave proves that advancing COVID-19 vaccination and waiting for vaccine coverage to meet “herd immunity” criteria need to be accompanied by ongoing implementation of non-pharmacological interventions to reduce the transmission rate of infection. During the two waves, the UK implemented three national lockdowns, which successfully reduced the number of infected people and mitigated the spread of COVID-19. The second lockdown was less stringent than the other two, and the mortality per million during the lockdown remained at a high level. Therefore, the implementation of a stricter lockdown can ensure the best control effect during the lockdown. In addition, all three lockdowns were characterized by mitigation strategy, all aimed at mitigating the surge in infections and did no emphasis on isolation and management of patients with mild symptoms, suspected cases, and close contacts. The UK is a typical country that adopts mitigation strategy in response to COVID-19. Although it can slow the spread of the pandemic in the short term and flatten the epidemiological curve, there is a risk that COVID-19 will rebound several times when herd immunity is insufficient.

## Data Availability

All data generated or analyzed during this study are included in this published article.
